# The optimal dose of dexmedetomidine as a 0.59% ropivacaine adjuvant for epidural anesthesia in great saphenous varicose vein surgery, based on hemodynamics and anesthesia efficacy: a randomized, controlled, double-blind clinical trial

**DOI:** 10.3389/fmed.2024.1426512

**Published:** 2024-07-24

**Authors:** Sisi Zeng, Xuechao Li, Hongchun Xu, Qin Ye, Zhaogang Li, Fangjun Wang

**Affiliations:** ^1^Department of Anesthesiology, Xinqiao Hospital, Chongqing, China; ^2^Affiliated Hospital of North Sichuan Medical College, Nanchong, China; ^3^First People’s Hospital of Chongqing Liangjiang New District, Chongqing, China; ^4^Zigong Fourth People's Hospital, Zigong, China; ^5^The People's Hospital of Leshan, Leshan, China

**Keywords:** dexmedetomidine, epidural anesthesia, hemodynamics, anesthetic effect, ropivacaine

## Abstract

**Objective:**

This study aimed to explore the optimal dose of dexmedetomidine as a 0.59% ropivacaine adjuvant for epidural anesthesia on perioperative hemodynamics and anesthesia efficacy in patients undergoing great saphenous varicose vein surgery.

**Methods:**

A total of 90 patients were randomly divided into three groups: 0.25 μg/kg dexmedetomidine combined with 0.59% ropivacaine epidural infusion group (ED_1_ group), 0.5 μg/kg dexmedetomidine combined with 0.59% ropivacaine epidural infusion group (ED_2_ group), and 0.75 μg/kg dexmedetomidine combined with 0.59% ropivacaine epidural infusion group (ED_3_ group). Hemodynamics, anesthesia efficiency, and adverse reactions were recorded.

**Main results:**

Compared with the ED_1_ group, the ED_2_ group had lower systolic blood pressure at T_1-3_ (T_1_, 95%CIs, 6.52–21.93, *p* < 0.001; T_2_, 95%CIs, 2.88–18.21, *p* = 0.004; T_3_, 95%CIs, 0.49–18.17, *p* = 0.035), and the diastolic blood pressure at T_1-2_ was decreased (T_1_, 95%CIs, 4.55–14.36, *p* < 0.001; T_2_, 95%CIs, 0.37–12.17, *p* = 0.033). Compared with the ED_2_ group, the ED_3_ group had higher systolic blood pressure at T_1-2_ (T_1_, 95%CIs, 5.90–21.46, *p* < 0.001; T_2_, 95%CIs, 2.07–17.55, *p* = 0.008) and higher diastolic blood pressure at T_1-3_ (T_1_, 95%CIs, 2.91–12.81, *p* = 0.001; T_2_, 95%CIs, 1.32–13.23, *p* = 0.011; T_3_, 95%CIs, 0.14–11.52, *p* = 0.043). Compared with the ED_2_ group, the heart rate was significantly decreased at T_1-4_ in the ED_3_ group (T_1_, 95%CIs, 2.25–15.72, *p* = 0.005; T_2_, 95%CIs, 2.35–13.82, *p* = 0.003; T_3_, 95%CIs, 0.50–9.79, *p* = 0.025; T_4_, 95%CIs, 1.46–10.36, *p* = 0.005). The myocardial oxygen consumption in all three groups was significantly decreased at each time point compared to T_0_ (*p* < 0.05 or < 0.001), and no significant between-group differences were detected (*P*>0.05). Compared with the ED_1_ group, the anesthesia efficiency of ED_2_ and ED_3_ groups was markedly enhanced, but the risk of bradycardia in ED_2_ and ED_3_ groups was dramatically increased (6 of 28 [21.4%] vs. 14 of 30 [46.7%] and 14 of 27 [51.9%], *p* = 0.023), one patient in the ED_3_ group experienced difficulty urinating, and remaining adverse reactions were mild in all three groups.

**Conclusion:**

A measure of 0.5 μg/kg dexmedetomidine is the optimal dose as a 0.59% ropivacaine adjuvant for epidural anesthesia in patients undergoing great saphenous varicose vein surgery.

**Clinical trial registration:**

http://www.chictr.org.cn/, registration number: ChiCTR2200060619.

## 1 Introduction

Epidural anesthesia is safer relatively than other anesthesia techniques, has fewer complications, can diminish urinary retention, prevent thrombosis, and improve postoperative recovery and early ambulation of patients compared with patients undergoing general anesthesia and spinal anesthesia (1–3). In particular, epidural anesthesia has an increased risk of a slow onset and incomplete blockage (4), which reduces patients’ satisfaction with epidural anesthesia to a certain extent. Therefore, to achieve expected anesthetic effect and avoid the toxicity of local anesthetic caused by increasing the dosage of local anesthetic, some researchers have proposed to add some local anesthetic adjuvants combined with local anesthetic in epidural anesthesia, in which dexmedetomidine has fewer adverse reactions than other local anesthetic adjuvants (clonidine, tramadol, fentanyl, sufentanil, etc.), and the anesthetic efficiency is remarkably ameliorated (5–8).

Dexmedetomidine is a highly selective α_2_-adrenergic receptor agonist that downregulates sympathetic nerve activity and maintains the “awake sedative” of arousal function, with anxiolytic, analgesic effects, and wide safety margins (9). Prior studies and our previous studies have revealed that the injection of dexmedetomidine 0.5 μg/kg as a local anesthetic adjuvant into epidural space synergistically ameliorates the impact of epidural anesthesia and is steadier in perioperative hemodynamics than intravenous dexmedetomidine patients, which is beneficial to perioperative patient management in epidural anesthesia (10–12). Observational data find that epidural infusion of dexmedetomidine has a conspicuous influence on hemodynamics (7). Nonetheless, a historical cohort study has reported that intraoperative use of dexmedetomidine exceeding 50 μg is highly likely to contribute to postoperative hypotension after the postanesthesia care unit (PACU), which considerably correlated with poor patient prognosis (13). The reason for this result may be related to the dose of epidural infusion dexmedetomidine (7, 13). However, to the best of our knowledge, there has been no literature report regarding the optimal recommended dose of epidural infusion dexmedetomidine that can ensure both the effectiveness of epidural anesthesia and remain stable hemodynamics in patients across the perioperative period. Past studies have demonstrated that epidural infusion of 0.5 μg/kg dexmedetomidine combined with local anesthesia can perform excellent epidural anesthesia efficiency and mild adverse reactions (5, 12). Therefore, we hypothesize that 0.5 μg/kg dexmedetomidine as epidural local anesthetic adjuvant would be the optimal recommended dose with excellent epidural effect, stable hemodynamics, and minor adverse effects. Accordingly, we took 0.5 μg/kg as the intermediate dose and added or subtracted 0.25 μg/kg to investigate preliminarily the effects of dexmedetomidine at diverse doses combined with ropivacaine epidural anesthesia on perioperative hemodynamics and anesthesia effects in patients, to provide a reference for the optimal recommended dose of dexmedetomidine as an adjuvant in clinical epidural anesthesia.

## 2 Materials and methods

### 2.1 Trial design

This prospective, randomized, double-blind, controlled clinical trial was conducted between 8 June 2022 and 30 November 2022 in patients undergoing elective saphenous vein peeling or planning surgery under epidural anesthesia. The enrollment of the first patient took place on 8 June 2022. This protocol was reviewed and approved by the Medical Ethics Committee of the Affiliated Hospital of North Sichuan Medical College (Ref. 2022ER073-1) and registered in the China Clinical Trial Registry on 5 June 2022 (http://www.chictr.org.cn/; registration number: ChiCTR2200060619). A written informed consent was obtained from all participants. All methods were performed in accordance with Consolidated Standards of Reporting Trials (14).

### 2.2 Participants

#### 2.2.1 Inclusion criteria

The inclusion criteria were as follows: (1) American Society of Anesthesiologists (ASA) grades I or II. (2) Patients scheduled for saphenectomy under epidural anesthesia are screened. (3) Age 18–65 years old, gender is not limited. (4) Height 140–180 cm and weight 40–80 kg. (5) No contraindications to epidural anesthesia. (6) Those who signed the informed consent form.

#### 2.2.2 Exclusion criteria

The exclusion criteria were as follows: (1) Patients with hypertension (systolic blood pressure>160 mmHg) or hypotension (systolic blood pressure<90 mmHg). (2) Patients with cardiac insufficiency and bradycardia (heart rate<50 beats per minute). (3) Patients allergic to dexmedetomidine injection and ropivacaine. (4) Long-term use of analgesics, sedatives, depressant drugs, adrenergic receptor antagonists, and agonists. (5) Liver and kidney insufficiency. (6) Patients with lower limb movement, hearing dysfunction, and mental system diseases.

#### 2.2.3 Withdrawal criteria

The withdrawal criteria were as follows: (1) Due to the failure of the epidural anesthesia block, change the anesthesia mode (such as general anesthesia surgery or subarachnoid block). (2) Those who change the puncture site due to anatomical variation. (3) Patients and their families automatically withdraw from the experiment. (4) Those who are allergic to experimental drugs unknown before surgery.

### 2.3 Randomization

This clinical trial was intended to adopt a completely random design method, 90 numbers were randomly assigned 1:1:1 to three groups according to the random number table, the drug formula corresponding to each number group was written on the back of the number card, and the card was sealed in an opaque envelope. When the patient entered the operating room, the nurse randomly selected an envelope and dispensed with medication according to the card prompt in the envelope.

### 2.4 Blinding method

When the patients entered the operating room, the nurse randomly selected an envelope, dispensed with the drug according to the card prompt in the envelope, and submitted it to the anesthesiologist of this trial after completion, and the anesthesiologist was blind of the patient’s grouping, saved the blind bottom file, and prepared emergency letters. After the trial was completed, the anesthesiologist reported the data back to the statistician, who analyzed the results. All participants, preoperative and postoperative follow-up assessors, and statisticians were blinded to the group allocation. Cohort and interventions were announced after trial termination and completion of data analysis.

### 2.5 Sample size

The sample size was calculated based on the results of our pilot study and the study by Bloor et al. (15). The standard deviation of the three groups was 11, and we assumed that the mean deviation of systolic blood pressure in the three groups is ≥10 mmHg as there is a difference. To achieve 95% power, a 0.05 two-sided significance level, and a 20% dropout rate for the test, a total of 90 cases (30 in each group) were needed, as calculated using PASS 2021.

### 2.6 Statistical methods

(1)The Shapiro–Wilk test was used to determine the distribution of the data: normal or skewed distribution. The continuous data of normal distribution were expressed by mean (standard deviation, SD), the non-normally distributed data were expressed as median (IQR), and categorical variables were represented by relative numbers (R) and percentage. One-way ANOVA was used for inter-group comparison; comparison at different time points was performed using two-way repeated-measures ANOVA. Bonferroni correction was used for post-event pairwise comparison. The Welch test was used for uneven variance, and the Tamhane test was used for post-event pairwise comparison. If the intra-group interaction effect was significant (*p* < 0.05), the simple-effect test was used for intra-group comparison, and Bonferroni correction was used for pairwise comparison. Kruskal–Wallis H-test was used for grade data comparison, and χ^2^ test or Fisher’s exact probability method was used for counting data. (2) SPSS 26.0 statistical software was used for statistical analysis. (3) A two-tailed *p*-value<0.05 was taken to indicate statistical significance.

### 2.7 Intervention

#### 2.7.1 Preoperative management

On the day before surgery, anesthesiologists involved in this study visited patients, informed them of anesthesia risks and accidents in the course of surgery, instructed patients to fast for 6–8 h before surgery and refrain from drinking for more than 2 h, explained the research process, and obtained the consent of patients and their families to sign anesthesia informed consent and research informed consent. The patients were also familiar with the visual analog scale (VAS) (16) and the Postoperative Anesthesia Satisfaction Scale (see Appendix).

#### 2.7.2 Intraoperative management

All enrolled patients received no premedication. Routine monitoring was conducted upon the patient’s arrival at the operation room, including heart rate (HR), electrocardiogram (ECG), non-invasive blood pressure (NBP), and saturation of hemoglobin with oxygen (SPO_2_). A fresh oxygen flow of 2 L/min was routinely given via a facial mask. Simultaneously, patients were preloaded with 20 mL·kg^−1^·h^−1^ of lactated Ringer’s solution. Epidural anesthesia procedure was performed in the left lateral decubitus position for all patients at L_2-3_ interspace, and the puncture was conducted by direct insertion method. We use the loss-of-resistance technique with physiological saline to identify the epidural space. The catheter was inserted into the epidural space up to 4–5 cm and secured there once it was confirmed that there was no blood or cerebrospinal fluid on negative aspiration. Then, the test dose of 3 mL 1.5% lidocaine (Lidocaine hydrochloride injection, 5 mL: 100 mg, Shiyao Yinhu Pharmaceutical Co, Ltd., China) was given to rule out the possibility of total spinal anesthesia. After 5 min, the ED_1_, ED_2_, and ED_3_ groups were given 0.25 μg/kg, 0.5 μg/kg, and 0.75 μg/kg dexmedetomidine (Dexmedetomidine hydrochloride injection, 2 mL:0.2 mg, Yangtze River Pharmaceutical [Group] Co, Ltd., China), 2 mL combined with 0.59% ropivacaine (Ropivacaine hydrochloride injection, 10 mL:100 mg, AstraZeneca AB, Sweden), and 15 mL epidural infusion, respectively, at a rate of 0.5 mL/s. T_10_ level in the bilateral pain anesthesia block plane was considered a successful anesthesia block. The baseline of BP and HR was defined as the average of three consecutive measurements at the time when patients arrived in the operating room in a supine position. Across the operation, the fluctuation range of blood pressure was maintained within plus or minus 20% of the basic value. Once hypotension occurred, defined as systolic blood pressure <90 mmHg, fluid therapy was performed firstly; if it did not ameliorate, 6 mg ephedrine was treated with repeated administration until recovery; and if the heart rate continued to be lower than 50 beats/min and exhibited a downward trend, treatment of 0.5 mg atropine could be administered, repeatedly if needed. Respiratory depression was defined as SPO_2_ < 90% and treated with a face mask oxygen inhalation and respiratory support if required. Patients with intraoperative VAS > 3 and who need analgesia were given epidural 3 mL of 1% ropivacaine as an additional local anesthetic. If the pain was not relieved, tramadol 2 mg/kg was injected intramuscularly. If there was no relief after 30 min, tramadol was added, and the maximum dose could be added to 200 mg. If VAS was still >3, it would be changed to general anesthesia to withdraw from the trial.

#### 2.7.3 Postoperative management

When the operation procedure was completed, patients were transferred to the ward. Adverse reactions within 24 h after surgery were recorded in regular follow-up. Ephedrine 6 mg was administrated to patients whose systolic blood pressure remained below 90 mmHg after surgery. After the operation, patients with persistent bradycardia [heart rate < 50 beats/min (17)] were given 0.5 mg atropine, if nausea and vomiting require medication, ondansetron 4 mg should be given, and patients with VAS > 3 and requiring analgesia were given tramadol 2 mg/kg. The above drugs can be repeated as necessary until symptoms resolve in accordance with the standard of clinical medication (lockout time of ephedrine and atropine administration: 5 min, lockout time of tramadol and ondansetron administration: 30 min).

### 2.8 Observation indicators

#### 2.8.1 Primary outcome parameters

Primary outcome parameters were as follows: systolic blood pressure (SBP) was documented before anesthesia (T_0_), at 5 min (T_1_), 15 min (T_2_), 30 min (T_3_), 1 h (T_4_), 2 h (T_5_), 4 h (T_6_), 6 h (T_7_), and 8 h (T_8_) after dexmedetomidine infusion.

#### 2.8.2 Secondary outcome parameters

Secondary outcome parameters were as follows: (1) Diastolic blood pressure (DBP) and heart rate (HR) were recorded before anesthesia (T_0_), at 5 min (T_1_), 15 min (T_2_), 30 min (T_3_), 1 h (T_4_), 2 h (T_5_), 4 h (T_6_), 6 h (T_7_), and 8 h (T_8_) after dexmedetomidine infusion. (2) Plasma norepinephrine (NE) concentration: 5 mL of venous blood was drawn at T_0_ and T_3-7_ and heparinized; blood samples were immediately centrifuged at a rate of 2000 r/min for 20 min, and 2 mL of supernatant was placed in a low-temperature freezer at −80°C for testing. Plasma NE concentration was determined by ELISA. (3) Anesthetic effect: the anesthesia plane was determined by the acupuncture pain disappearance method (18). The onset time of bilateral epidural pain anesthesia block (it was defined as the time from the start of epidural infusion to a T_10_ sensory block level being achieved), the time for Ramsay score (18) to reach 4 after epidural dexmedetomidine administration (sleep state). The highest block level, the time of anesthesia and analgesia (the time from epidural puncture injection of local anesthetics to postoperative pain (VAS>3 points), modified Bromage score (19) (when the block takes effect) was used to assess the degree of lower extremity motor blockade and the recovery time of lower extremity muscle strength (Bromage>4 points), and the patient’s postoperative anesthesia satisfaction was evaluated with the Anesthesia Satisfaction Scale (20). (4) The myocardial oxygen consumption (MVO_2_) during anesthesia T_0-8_ in three groups was evaluated (21). (5) Ramsay sedation score was evaluated at T_0-5_ of anesthesia patients in the three groups. (6) Visual analog scale (VAS) was used to examine the analgesic effect in three groups of anesthetized patients at T_5-8_. (7) Adverse reactions: the occurrences of adverse reactions [hypotension, bradycardia, dizziness, nausea and vomiting, dry mouth, chills, respiratory depression (SPO_2_ < 90% or respiratory rate < 10 breaths/min) and 24-h postoperative amnesia, etc.] in three groups of patients were documented. The occurrences of dizziness were noted every 2 h within 24 h after surgery. (8) Others: general information about the research subjects (age, weight, etc.), operation time, total amount of intraoperative fluid replacement, intraoperative blood loss, additional local anesthetics, analgesic drugs, and intraoperative vasoactive drug dosage were recorded.

## 3 Results

### 3.1 Participants and loss to follow-up

**Figure 1:**
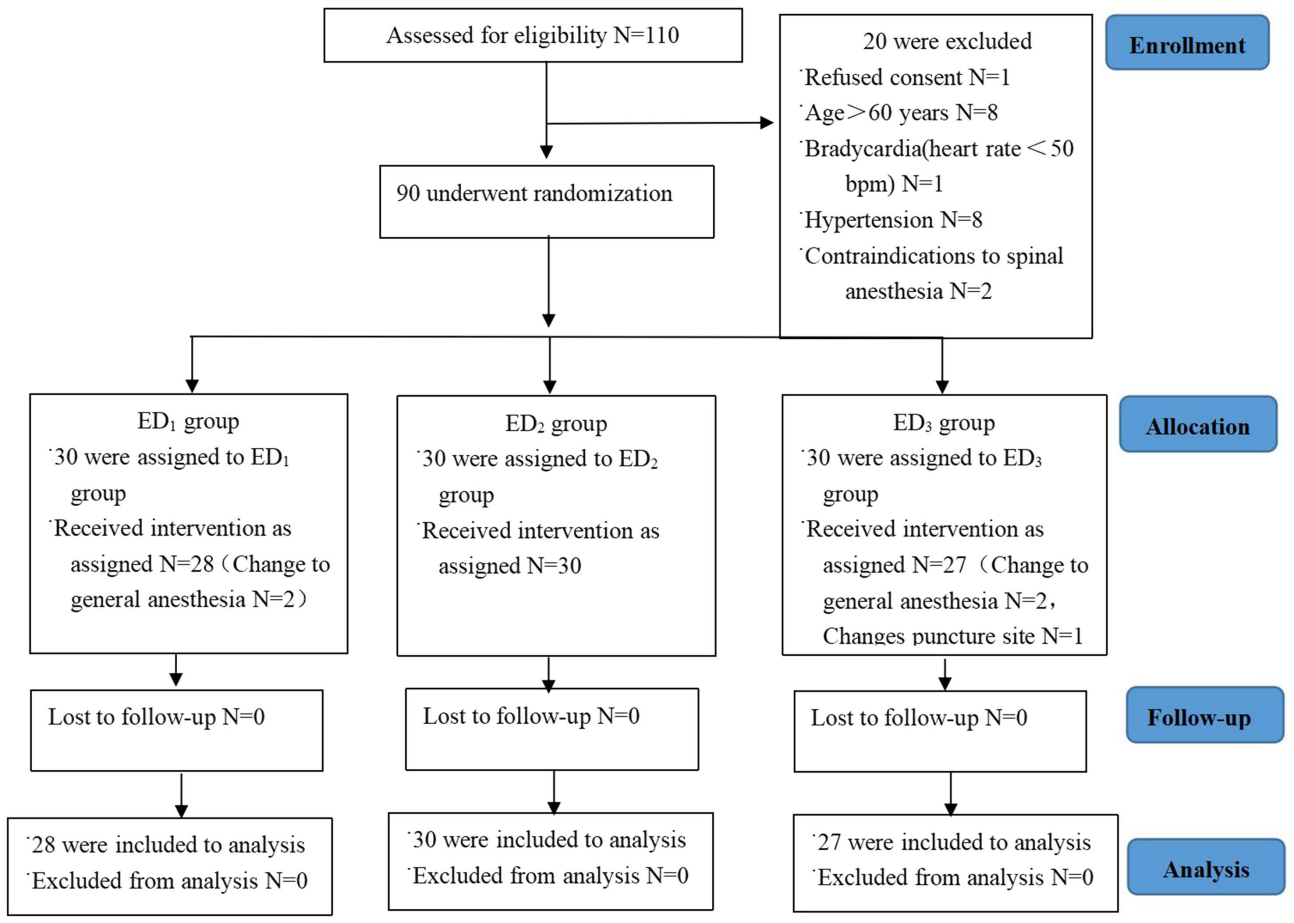
CONSORT study flow diagram. Flowchart epitomizing inclusion, allocation, and analysis. Abbreviations: ED_1_ group, 0.59% ropivacaine combined with 0.25 μg/kg dexmedetomidine epidural infusion group; ED_2_ group, 0.59% ropivacaine combined with 0.5 μg/kg dexmedetomidine epidural infusion group; ED_3_ group, 0.59% ropivacaine combined with 0.75 μg/kg dexmedetomidine epidural infusion group.

Between June 2022 and October 2022, a total of 110 patients were recruited for evaluation, of which 20 patients did not meet the inclusion criteria and were excluded from this study. Among them, 5 patients withdrew from the trial, no patient was lost to follow-up, and a randomized analysis of 85 patients undergoing surgery was conducted (ED_1_ group N = 28, ED_2_ group N = 30, and ED_3_ group N = 27) (Figure 1).

### 3.2 Demographic characteristics and intraoperative general information

No significant between-group differences were detected for any of the general data, including the operation time, infusion volume, blood loss, and additional local drugs, and three groups had similar baseline characteristics (*p* > 0.05) (Table 1).

**Table 1:** General data comparison among the three groups [ $\bar{x}$ *± s*, median (Q1,Q3), and relative numbers (*R*)].

	ED_1_ group (*n* = 28)	ED_2_ group (*n* = 30)	ED_3_ group (*n* = 27)	*F/H/χ^2^*	*p*-value
Age (yr)	55.7 ± 6.5	53.5 ± 7.3	53.5 ± 7.3	0.918	0.403
Gender (male/female)	13/15	17/13	12/15	0.986	0.611
ASA(I/II)	15/13	16/14	13/14	0.208	0.901
Body weight (kg)	66.3 ± 8.7	64.8 ± 10.2	64.6 ± 8.4	0.285	0.753
Height (cm)	162.0 ± 6.0	162.8 ± 8.3	163.6 ± 7.4	0.324	0.725
Operation time (min)	111.1 ± 19.9	107.6 ± 30.5	110.2 ± 21.	0.160	0.583
Infusion volume (mL)	1139.3 ± 166.3	1100.0 ± 249.1	1159.3 ± 229.1	0.548	0.580
Blood loss (mL)	89.8 ± 23.5	93.3 ± 25.1	86.1 ± 22.7	0.651	0.524
Use of vasoactive drug (yes/no)	3/25	4/26	6/21	1.543	0.462
Use of antiemetic (yes/no)	0/28	0/30	0/27	0.000	1.000
Additional local anesthetics and analgesic drugs (yes/no)	0/28	0/30	0/27	0.000	1.000

### 3.3 Outcomes analysis

#### 3.3.1 Hemodynamics

**Figure 2:**
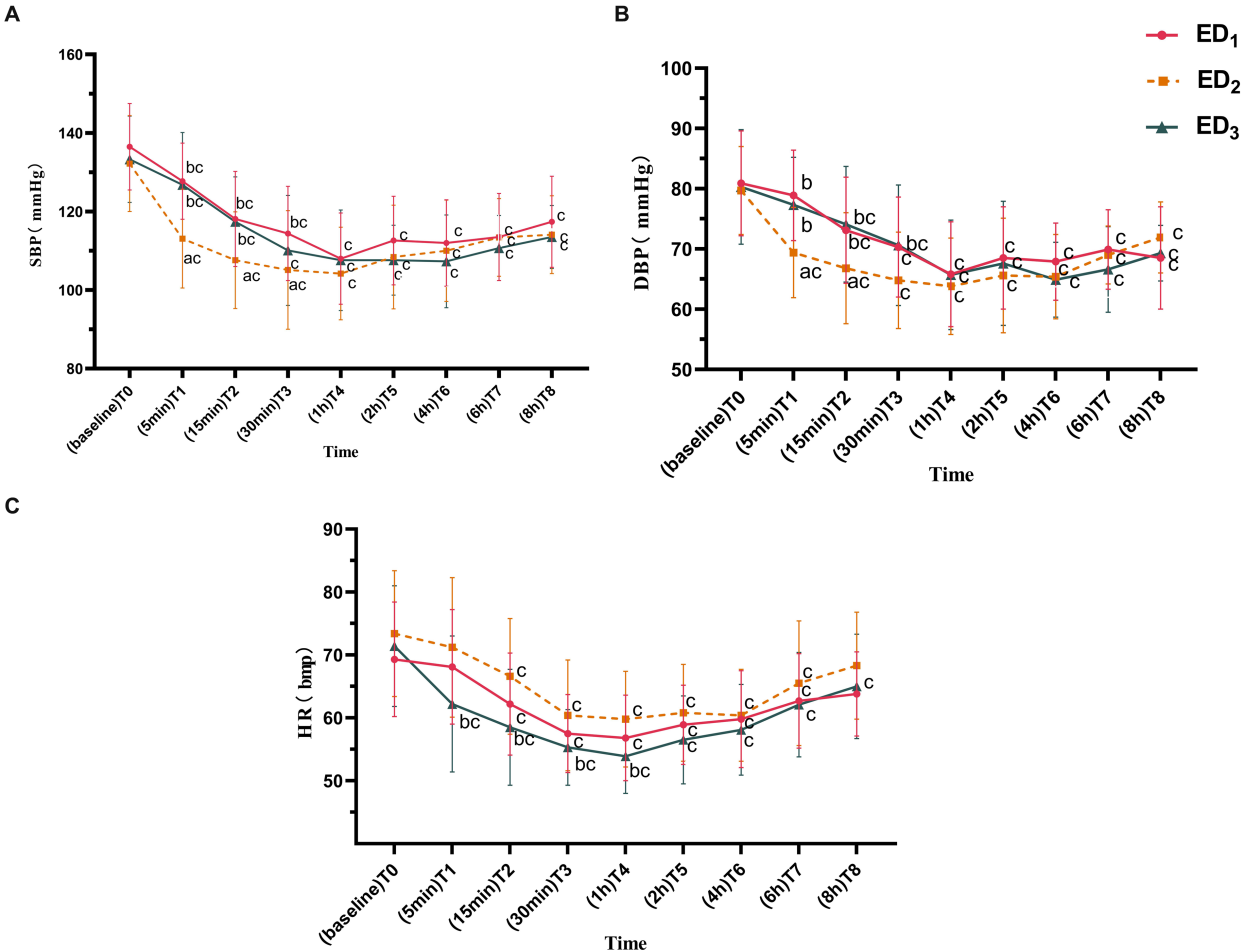
**(A)** Systolic blood pressure of the ED_1_, ED_2_, and ED_3_ groups at baseline, 5 min, 15 min, 30 min, 1 h, 2 h, 4 h, 6 h, and 8 h after administration. **(B)** Diastolic blood pressure of the ED_1_, ED_2_, and ED_3_ groups at baseline, 5 min, 15 min, 30 min, 1 h, 2 h, 4 h, 6 h, and 8 h after administration. **(C)** Heart rate of the ED_1_, ED_2_, and ED_3_ groups at baseline, 5 min, 15 min, 30 min, 1 h, 2 h, 4 h, 6 h, and 8 h after administration. SBP, systolic blood pressure; DBP, diastolic blood pressure; HR, heart rate. ED_1_ group, 0.59% ropivacaine combined with 0.25 μg/kg dexmedetomidine epidural infusion group; ED_2_ group, 0.59% ropivacaine combined with 0.5 μg/kg dexmedetomidine epidural infusion group; ED_3_ group, 0.59% ropivacaine combined with 0.75 μg/kg dexmedetomidine epidural infusion group. ^a^*p* < 0.05, vs. ED₁ group; ^b^*p* < 0.05, vs. ED_2_ group; ^c^*p* < 0.05:vs. T_0_, both comparisons were corrected by Bonferroni.

Compared with the ED_1_ group, the SBP in the ED_2_ group was lower at T_1-3_ (T_1_, 95%CIs, 6.52–21.93, *p* < 0.001; T_2_, 95%CIs, 2.88–18.21, *p* = 0.004; T_3_, 95%CIs, 0.49–18.17, *p* = 0.035), and there was no significant difference in the ED_3_ group at each time point; compared with the ED_3_ group, the ED_2_ group had a lower SBP at T_1-2_ (T_1_, 95%CIs, 5.90–21.46, *p* < 0.001; T_2_, 95%CIs, 2.07–17.55, *p* = 0.008). Compared to T_0_, the SBP at T_1-8_ in ED_1_, ED_2_, and ED_3_ groups was significantly lower (*P*<0.05 or <0.001) (Figure 2A). Compared with the ED_1_ group, the ED_2_ group had lower DBP at T_1-2_ (T_1_, 95%CIs, 4.55–14.36, *p* < 0.001; T_2_, 95%CIs, 0.37–12.17, *p* = 0.033), and there exists no significant difference in ED_3_ group at various time points; the DBP was higher in the ED_3_ group at T_1-3_ compared with the ED_2_ group (T_1_, 95%CIs, 2.91–12.81, *p* = 0.001; T_2_, 95%CIs, 1.32–13.23, *p* = 0.011; T_3_, 95%CIs, 0.14–11.52, *p* = 0.043). Compared to T_0_, the DBP in the ED_1_ group was lowered significantly at T_2-8_ (*p* < 0.001), while in the ED_2_ group, it decreased observably at T_1-8_ (*p* < 0.05 or < 0.001), and in the ED_3_ group, it reduced significantly at T_2-8_ (*p* < 0.05 or < 0.001) (Figure 2B). Compared with the ED_1_ group, there was no remarkable difference in the HR at each time point between the ED_3_ and ED_2_ groups (*p* > 0.05). Compared with ED_2_ group, the HR of ED_3_ group significantly decreased at T_1-4_ (T_1_, 95%CIs, 2.25–15.72, *p* = 0.005; T_2_, 95%CIs, 2.35–13.82, *p* = 0.003; T_3_, 95%CIs, 0.50–9.79, *p* = 0.025; T_4_, 95%CIs, 1.46–10.36, *p* = 0.005); compared to T_0_, the HR dramatically declined at T_2-7_ in the ED_1_ and ED_2_ groups and decreased at T_1-8_ in the ED_3_ group (*p* < 0.05 or < 0.001) (Figure 2C).

**Figure 3:**
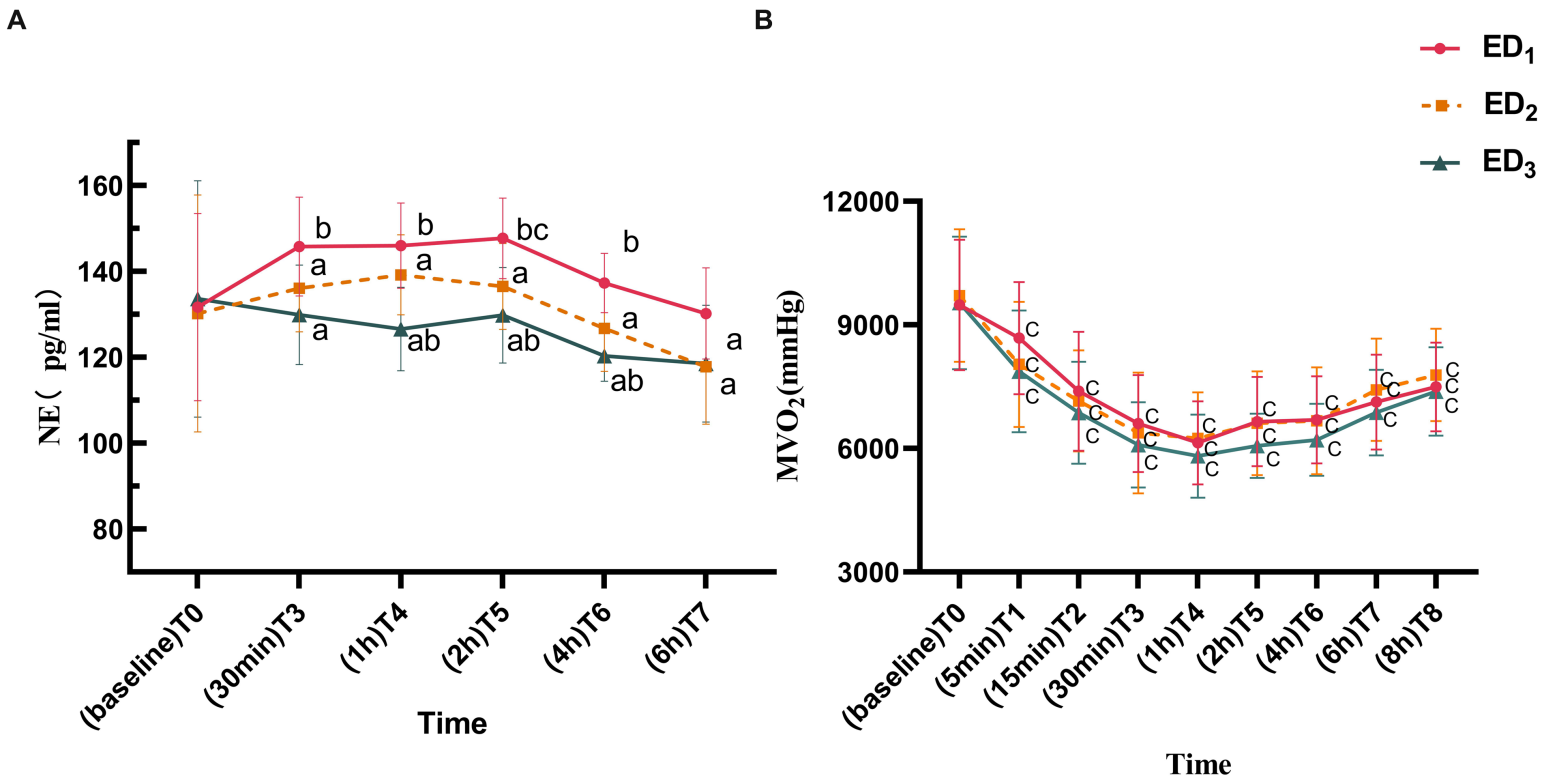
**(A)** Norepinephrine of ED_1_, ED_2_, and ED_3_ groups at baseline, 30 min, 1 h, 2 h, 4 h, and 6 h after administration. **(B)** Myocardial oxygen consumption of the ED_1_, ED_2_, and ED_3_ groups at baseline, 5 min, 15 min, 30 min, 1 h, 2 h, 4 h, 6 h, and 8 h after administration. NE, norepinephrine; MVO₂, myocardial oxygen consumption. ED_1_, group, 0.59% ropivacaine combined with 0.25 μg/kg dexmedetomidine epidural infusion group; ED_2_ group, 0.59% ropivacaine combined with 0.5 μg/kg dexmedetomidine epidural infusion group; ED_3_ group, 0.59% ropivacaine combined with 0.75 μg/kg dexmedetomidine epidural infusion group. ^a^*p* < 0.05, vs. ED_1_ group; ^b^*p* < 0.05, vs. ED_2_ group; ^c^*p* < 0.05:vs. T_0_, both comparisons were corrected by Bonferroni.

Compared with the ED_1_ group, both ED_2_ and ED_3_ groups showed a conspicuous reduction in NE concentration at T_3-7_ (*p* < 0.05 or < 0.001); compared with the ED_2_ group, the NE concentration in ED_3_ group significantly decreased at T_4-6_ (*p* < 0.05 or < 0.001). Compared to T_0_, the NE concentration in the ED_1_ group was markedly elevated at T_5_ (*p* = 0.026), while there was no statistically significant difference between ED_2_ and ED_3_ groups at each time point (*p* > 0.05) (Figure 3A). MVO_2_ was significantly lower in all three groups at each time point compared to T_0_ (*p* < 0.05 or < 0.001), and there was no statistically significant difference among the groups (*p* > 0.05) (Figure 3B).

#### 3.3.2 Anesthetic effect

**Figure 4:**
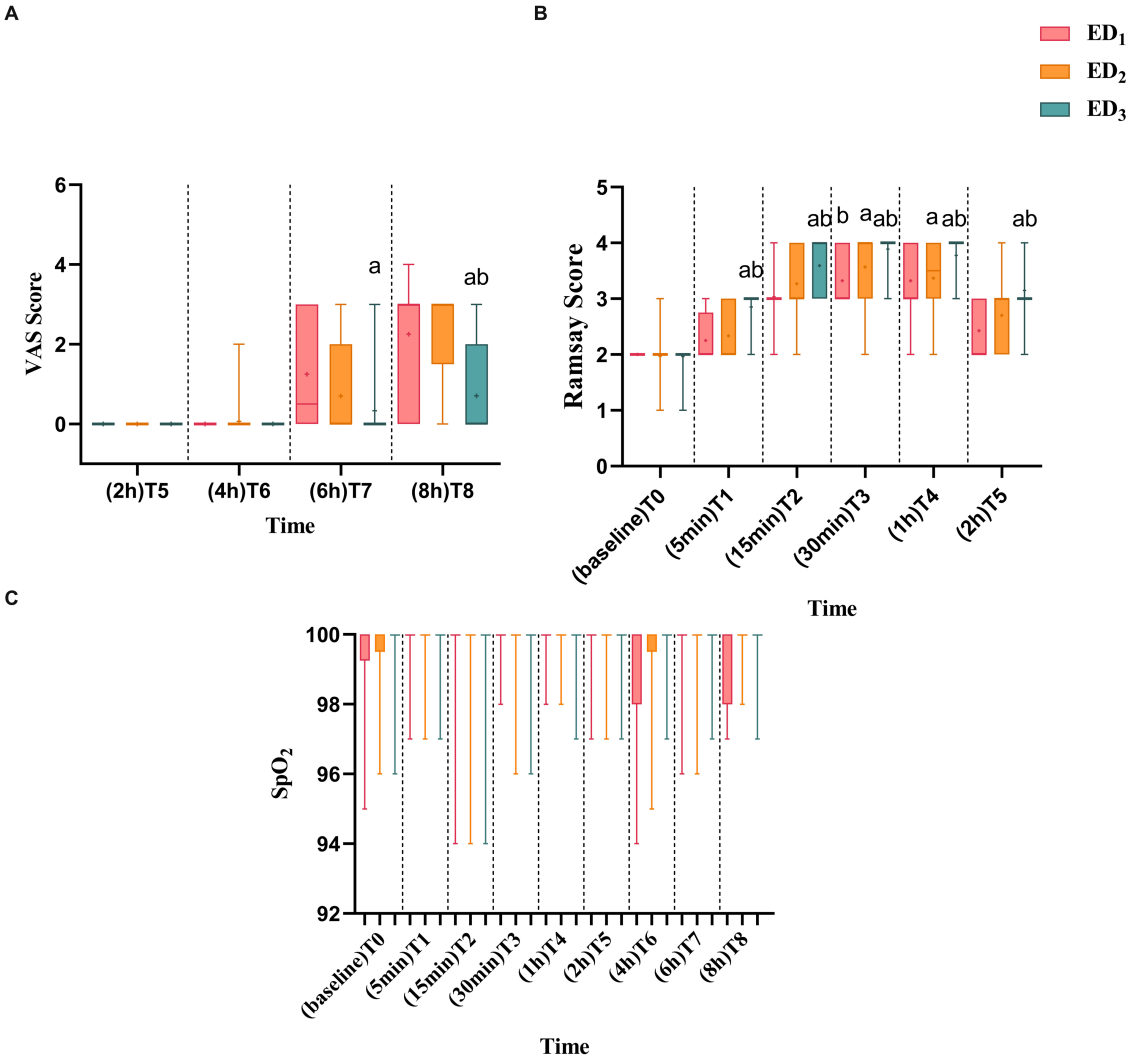
**(A)** ED_1_, ED_2_, and ED_3_ groups were scored with VAS scores at 2 h, 4 h, 6 h, and 8 h after administration. **(B)** ED_1_, ED_2_, and ED_3_ were scored with Ramsay scores at baseline, 5 min, 15 min, 30 min, 1 h, and 2 h after administration. **(C)** Blood oxygen saturation of ED_1_, ED_2_, and ED_3_ groups at baseline, 5 min, 15 min, 30 min, 1 h, 2 h, 4 h, 6 h, and 8 h after administration. VAS, visual analog scale; S_P_O_2_, blood oxygen saturation, ED_1_ group, 0.59% ropivacaine combined with 0.25 μg/kg dexmedetomidine epidural infusion group: ED_2_ group, 0.59% ropivacaine combined with 0.5 μg/kg dexmedetomidine epidural infusion group; ED_3_ group, 0.59% ropivacaine combined with 0.75 μg/kg dexmedetomidine epidural infusion group. ^a^*p* < 0.05, vs. ED_1_ group; ^b^*p* < 0.05, boxes indicate the median with the 25th and 75th percentiles (interquartile range), whisker caps represent the minimum and maximum values, and + represents the average.

Compared with the ED_1_ group, the Ramsay score was significantly increased in the ED_2_ group at T_3_ (*p* = 0.032), whereas the Ramsay score of the ED_3_ group observably declined at T_1-5_ (*p* < 0.05 or < 0.001). Moreover, the Ramsay score was significantly lower in the ED_3_ group at T_1-5_ (*p* < 0.05 or < 0.001) compared with the ED_2_ group (Figure 4A). Compared with the ED_1_ group, there was no statistically obvious discrepancy in VAS scores between the ED_2_ group at various time points, yet the ED_3_ group showed a noteworthy decrease in VAS scores at T_7-8_ (T_7_, median [IQR], 0.5 [3] vs. 0.0 [0.0], *p* = 0.003; T_8_, 3.0 [3.0] vs. 0.0 [2.0], *p* < 0.001) and compared with the ED_2_ group, the VAS score in ED_3_ group was considerably lower at T_8_ (median [IQR], 3 [1.5] vs. 0.0 [2.0], *p* < 0.001) (Figure 4B). There was no statistically significant difference in oxygen saturation among three groups (Figure 4C).

As the Ramsay score of four patients in the ED_1_ group and one patient in the ED_2_ group did not reach 4, considering the accuracy and reliability of the data, to avoid the bias caused by the data, we did not exclude these 5 data points. Instead, we defined the time of patients whose Ramsay score did not reach 4 as 40 min longer than any other patients. The ED_3_ group had a higher highest block plane (T_4_ vs. T_6_ & T_6_, *p* < 0.001), faster anesthesia onset time (7.0 [6.0, 8.0] min vs. 11.0 [10.0, 13.0] & 8.0 [7.0, 9.0] min, *p* < 0.001), a faster time when the Ramsay score reached 4 (15.0 [15.0, 20.0] min vs. 30.0 [25.0, 40.0] min & 23.0 [15.0, 30.0] min, *p* < 0.001), and longer analgesic time than the ED_1_ and ED_2_ groups ([439.9 ± 69.0] min vs. [341.0 ± 75.8] min & [392.0 ± 68.7] min, *p* < 0.001). Compared with the ED_2_ group, the ED_1_ and ED_3_ groups had higher motor block scores, and the ED_1_ group had longer anesthesia onset time, time when Ramsay score reached 4, analgesia time, and lower limb muscle strength recovery time (*p* < 0.05 or < 0.001). Compared to the ED_1_ group, the recovery time of lower limb muscle strength in the ED_3_ group was prolonged (95%CIs, 98.30–21.96, *p* = 0.002) (Table 2).

**Table 2:** Comparison of anesthesia effects among the three groups [ $\bar{x}$ *± s*, median (Q1,Q3), and relative numbers (*R*)].

	Maximum sensory block level T4/T6/T8/T10	Motor block score	Onset time of sensory block at T10 (min)	Time of Ramsay ≥4 (min)	Time of analgesia (min)	Recovery time of lower limb muscle strength (min)	Patient satisfaction with anesthesia
ED_1_ group (n = 28)	1/13/12/2	5.0(4.05.0)^b^	11.0(10.0,13.0)^b^	30.0(25.0,40.0)^b^	341.0 ± 75.8^b^	287.6 ± 15.6^b^	7.5(6.3,8.0)
ED_2_ group (*n* = 30)	0/19/11/0	4.0(4.04.0)	8.0(7.0,9.0)^a^	23.0(15.0,30.0)^a^	392.0 ± 68.7^a^	329.0 ± 10.4^a^	7.0(7.0,8.0)
ED_3_ group (*n* = 27)	15/10/2/0^ab^	5.0(4.0,5.0)^b^	7.0(6.0,8.0)^ab^	15.0(15.0,20.0)^ab^	439.8 ± 69.0^ab^	347.7 ± 14.0^a^	8.0(7.0,8.0)
*F/H*	28.594	14.100	45.739	33.592	13.245	5.183	2.585
*P*	<0.001	0.001	<0.001	<0.001	<0.001	0.008	0.275

^a^*p* < 0.05, vs ED_1_ group; ^b^*p* < 0.05, vs ED_2_ group, both comparisons were corrected by Bonferroni.

#### 3.3.3 Adverse reactions

Regarding the adverse reactions, the incidence of sinus bradycardia in ED_2_ and ED_3_ groups was observably elevated compared to the ED_1_ group (6 of 28 [21.4%] *VS* 14 of 30 [46.7%] & 14 of 27 [51.9%], *p* = 0.023), and the time to first urination was extended in the ED_3_ group compared with the ED_1_ and ED_2_ groups ([447.5 ± 19.2] min vs. [355.0 ± 13.0] min & [330.8 ± 13.1] min *P*<0.001). Furthermore, one patient in the ED_3_ group experienced difficulty urinating and finally had a urinary catheter installed. There was no statistically noteworthy discrepancy in the remaining adverse reactions. Among them, there were 2 of 28 [7.1%], 3 of 30 [10%], and 4 of 27 [14.8%] patients of hypotension (SBP <90 mmHg) in the ED_1_, ED_2_, and ED_3_ groups, respectively. Dizziness occurred in 1 of 28 [3.6%], 7 of 30 [23.3%], and 6 of 27 [22.2%], respectively. Dry mouth occurred in 2 of 28 [7.1%], 3 of 30 [10%], and 4 of 27 [14.8%], respectively (Table 3).

**Table 3:** Comparison of intraoperative and postoperative adverse events among the three groups [*n_1_*(%), $\bar{x}$ *± s*].

	Hypotension	Bradycardia	Dizziness	Nausea and vomiting	Dry mouth	Respiratory depression	24-h postoperative amnesia	Vasoactive drug use	First urination time
ED_1_ group (n = 28)	2(7.1)	6 (21.4)^b^	1(3.6)	0 (0)	2 (7.1)	0 (0)	0 (0)	4(14.3)	355.0 ± 13.0
ED_2_ group (*n* = 30)	3(10)	14 (46.7)^a^	7(23.3)	1(3.3)	3 (10)	0 (0)	0 (0)	5(16.7)	330.8 ± 13.1
ED_3_ group (*n* = 27)	4(14.8)	14(51.9)^a^	6(22.2)	2(7.4)	4(14.8)	0 (0)	0 (0)	6(22.2)	447.5 ± 19.2^ab^
*F/X^2^*	0.906	7.500	5.539	1.975	0.906	0.000	0.000	0.626	16.152
*P*	0.685	0.023	0.066	0.413	0.685	1.000	1.000	0.773	<0.001

*n*_1_: Number of cases of each adverse reaction, ^a^*p* < 0.05 vs ED_1_ group; ^b^*p* < 0.05 vs ED_2_ group; both comparisons were corrected by Bonferroni.

## 4 Discussion

This study observed that 0.59% ropivacaine co-administered 0.75 μg/kg dexmedetomidine was associated with more stable hemodynamics rather than that of 0.25 μg/kg and 0.5 μg/kg dexmedetomidine groups. While its motor block time was also relatively prolonged, it increased the risk of urinary retention, although there is no statistical significance. Compared with 0.25 μg/kg dexmedetomidine, 0.5 μg/kg and 0.75 μg/kg epidural infusion anesthesia were more effective, but the occurrence of bradycardia increased. The adverse reactions of dexmedetomidine at all three doses were mild.

Dexmedetomidine has a protective effect on cerebral nerves and reverses the neurotoxicity of local anesthetic, indicating that direct exposure of nerve roots to clinical doses of dexmedetomidine is secure (22–24). Early studies (7, 25–27) have proposed that epidural infusion of dexmedetomidine can suppress the perioperative hemodynamics of patients to a certain extent, which is consistent with the result of this experiment. In this research, we detected that the perioperative SBP, DBP, and HR in the three groups decreased to varying degrees compared to baseline, and the trend was roughly the same. They all descended to the lowest point approximately 1 h after epidural infusion of dexmedetomidine and then gradually ascended with U-shaped response curves to blood pressure and heart rate (Figures 2A–C). The decline of perioperative hemodynamics in the three groups was primarily on account of the superposition of sympathetic nerve suppression induced by epidural anesthesia and the pharmacological effects of dexmedetomidine itself (28). On the one hand, epidural infusion of dexmedetomidine can promptly block the spontaneous firing rate of neurons, subsequently inhibit sympathetic tone by binding to α_2_-adrenergic receptor in the dorsal horn of the spinal cord, and depress the release of norepinephrine, resulting in the inhibition of circulation (29). On the other hand, the highly fat-soluble dexmedetomidine has high meningeal penetration. After epidural administration, it can easily diffuse to the spinal cord and brain through the dural sleeve, acting on the rostral ventrolateral medulla (RVLM, cardiovascular regulatory center), downregulating the neuronal activity of RVLM, thereby causing a lowering in central blood pressure and heart rate in turn (30–33). Nonetheless, this downregulation in blood pressure and bradycardia can be relieved with vasoactive drug treatment.

Although the hemodynamic trends were similar among the three groups, they were distinct in the influence of dexmedetomidine epidural infusion with various doses on hemodynamics among the three groups. Compared to 0.5 μg/kg dexmedetomidine epidural infusion group, 0.25 μg/kg and 0.75 μg/kg dexmedetomidine epidural infusion groups showed a smaller decrease in blood pressure, and there was a considerably significant discrepancy (*p* < 0.007) at 5 and 15 min of epidural infusion (Figures 2A,B), while 0.75 μg/kg dexmedetomidine had a lower descend than 0.5 μg/kg dexmedetomidine in heart rate at 5 min, 15 min, 30 min, and 1 h after epidural infusion (*p* < 0.03) (Figure 2C).

Research has illustrated that the impact of dexmedetomidine on hemodynamics exists a dose-dependent characteristic (34). By depressing the release of norepinephrine, dexmedetomidine to some extent suppresses perioperative hemodynamics in patients. As the dose of dexmedetomidine increases, the concentration of norepinephrine released into the blood declines, which is in line with the tendency of changes in serum norepinephrine concentration measured in our experiment among the three groups (Figure 3A). The patient’s blood pressure and heart rate are correlated with the dose of dexmedetomidine, which exhibits a dose-dependent decrease, while blood pressure manifests as a dimorphic change (34–36). Early epidural administration of high-dose dexmedetomidine can directly stimulate vascular smooth muscle α_2_ receptors to produce a transient hypertensive response, with a reflex descent in heart rate, followed by a decline in blood pressure without reflex tachycardia, and the explanation for this biphasic reaction may be that: (1) The rapid binding of dexmedetomidine to vascular α_2_ receptors induces initial peripheral vascular constriction, which subsequently spreads to the central nervous system, suppressing vasomotor centers and plasma norepinephrine level (37). (2) The sympathetic inhibitory effect of dexmedetomidine may also lower blood pressure by attenuating the secondary effect of homeostatic cardiovascular response (that is, under normal physiological conditions, lower blood pressure will reflexively elevate heart rate, thereby increasing cardiac output and raising blood pressure) (13). Since epidural anesthesia itself can downregulate sympathetic nerve activity and the higher dose of dexmedetomidine epidural infusion can effectively reduce the ED_95_ of ropivacaine (5), the concentration of ropivacaine in this research is fixed, which undoubtedly enhances the efficacy of ropivacaine, thus amplifying the inhibitory effect of epidural anesthesia on sympathetic nerve activity. The sympathetic nerve inhibition of dexmedetomidine overlaps with the impact of epidural anesthesia, and patients in the higher dose group throughout the perioperative period may not be able to effectively produce this secondary effect, which is compensatory tachycardia attenuation.

Epidural administration of dexmedetomidine can directly act on the spinal cord and redistribute it to the brainstem through systemic absorption, downregulating sympathetic nervous system activity and releasing norepinephrine, causing a temporary reduction in blood pressure, but this consequence can lead to a compensatory elevate in heart rate through antisympathetic excitatory effect, resulting in a gradual rise in blood pressure (34, 37, 38). This may explain why the blood pressure reduction in the high-dose group of 0.75 μg/kg dexmedetomidine combined with 0.59% ropivacaine epidural infusion was less than that in the medium dose of 0.5 μg/kg dexmedetomidine epidural infusion group in this study, and there was a statistical difference at 5 min and 15 min after epidural infusion (Figures 2A,B, *p* < 0.02), while the heart rate remained lower than 0.5 μg/kg dexmedetomidine epidural infusion group. The heart rate was statistically remarkable at 5 min, 15 min, 30 min, and 1 h after epidural infusion (Figure 2C, *p* < 0.03).

In this research, we also observed that the epidural infusion of dexmedetomidine at three doses could effectively reduce the perioperative myocardial oxygen consumption of patients over the perioperative period, without significant adverse reactions such as oxygen desaturation and respiratory depression (Figures 3B, 4C, Table 3). This phenomenon is strongly associated with the pharmacological impact of dexmedetomidine and the sympathetic nerve inhibitory effect produced by epidural anesthesia itself. The reduction of myocardial oxygen consumption can alleviate the burden of the heart, decline cardiac accidents, and is particularly beneficial to patients with heart disease such as coronary heart disease.

Dexmedetomidine combined with ropivacaine epidural anesthesia exerts a synergistic effect that can significantly improve epidural efficacy, which is in line with the result of this experiment (5, 7, 39). In our study, we discovered that the effect of epidural anesthesia was dose-dependent on the dose of dexmedetomidine. Compared to 0.25 μg/kg and 0.5 μg/kg of dexmedetomidine, 0.75 μg/kg of dexmedetomidine as an adjuvant to ropivacaine for epidural anesthesia (ED_3_ group) could conspicuously shorten the onset time of anesthesia, elevate the highest blocking plane, prolong the analgesic time, and shorten the time when Ramsay score reached 4 (Table 2). Interestingly, despite epidural efficacy was dose-dependent with dexmedetomidine dose, the degree of motor block in the 0.75 μg/kg dexmedetomidine group is lower than that in the 0.5 μg/kg group (ED_3_ vs. ED_2_, 5.0 [4.0, 5.0] vs. 4.0 [4.0, 4.0], *p* < 0.001). This may be highly correlated with our method of measuring motor blocks. We stipulate that when the bilateral pain level reaches T_10_, the degree of motor block in patients is measured. As the onset time of anesthesia in the higher dose group (0.75 μg/kg group) was shortened, the motor block of patients was not completely achieved, thus leading to the “illusion” that the degree of motor block of patients in the higher dose group was lower, which seems to be confirmed by the duration of motor block recorded in this experiment. We found that among the three groups, the motor block time was significantly extended more in a relatively higher dose dexmedetomidine group (ED_2_ and ED_3_) compared to the lower dose group (ED_1_ group) (*p* = 0.008) (Table 2). We believe that the prolongation of the motor block time is obviously not conducive to early postoperative ambulation of patients and acceleration of recovery. Meanwhile, we proposed that the duration of motor blockade seems to have a capping effect as the dose of local anesthetic adjuvant dexmedetomidine elevates. From Table 2, we discover that compared with the 0.5 μg/kg dexmedetomidine group, the analgesic time of 0.75 μg/kg dexmedetomidine was extended by approximately 40 min (*p* = 0.013), while the motor block time was only extended by less than 20 min (*p* = 0.325). Of course, further experimental evidence is needed to confirm this hypothesis. Nevertheless, previous studies (5) and our prior studies have confirmed that epidural infusion of dexmedetomidine can obviously prolong the motor block time of patients compared to simple epidural infusion of ropivacaine (0.59% ropivacaine group vs. 0.59% ropivacaine+0.5 μg/kg dexmedetomidine group, [249.0 ± 54.9] min vs. [327.8 ± 58.7] min, *p* < 0.001). Undoubtedly, this will lower the patient’s satisfaction with anesthesia to a certain extent.

However, it is worth noting that the concentration of ropivacaine performed in this experiment is fixed, and when we take dexmedetomidine with distinct doses as ropivacaine adjuvant, this undoubtedly alters the ED_95_ of ropivacaine in epidural anesthesia. Previous studies have revealed that high-dose ropivacaine (0.75%) epidural infusion can affect motor and sensory pathways in the spinal cord, reducing the amplitude of motor-evoked potential; when dexmedetomidine is administered as an adjuvant for ropivacaine epidural anesthesia, it can lower the effective concentration of ropivacaine by approximately 25% (5, 40). This indicates that in the higher dose group, we may only need a concentration of ropivacaine much less than 0.59% to achieve the same anesthetic effect, while lower concentrations of ropivacaine may shorten the patient’s motor block time and reduce the degree of the block (i.e., the effect of motor block separation), so as to satisfy patient’s early mobilization and prevent postoperative complications such as venous thrombosis, but this requires further research. Hence, at present, we need to weigh the advantages against the disadvantages of dexmedetomidine as a local anesthetic adjuvant for epidural anesthesia in prolonging motor block time of patients in clinical work.

In epidural anesthesia, the patients are awake and patients are prone to nervous anxiety, which leads to increased sympathetic nerve activity and myocardial oxygen consumption. In this experiment, epidural infusion of dexmedetomidine provided excellent sedation and analgesic effects and reduced myocardial oxygen consumption in patients (Figures 4A,B), which had also been confirmed in past studies (5, 41, 42). The satisfied sedative and analgesic effect of dexmedetomidine synergistically enhanced patient satisfaction with anesthesia (Table 2). In this study, only the incidence of sinus bradycardia was statistically different among the three groups in adverse reactions [ED_1_ vs. ED_2_ & ED_3_, 21.4% vs. 41.7% & 51.9%, *p* = 0.023]. Meanwhile, it should be noted that one patient in the ED_3_ group had urinary retention, which may be consistent with the enhancement of anesthetic effect of ropivacaine by the higher dose of dexmedetomidine. This would be probably avoided if the ropivacaine concentration was reduced to the minimum effective dose (i.e., ropivacaine ED_95_ at 0.75 μg/kg dexmedetomidine combined). It is of great significance that this study is not designed to compare side effect profiles so it would limit the strength of the conclusions for side effect differences or similarities between groups. However, this adverse reaction also reminds us that it is crucial to explore the ED_95_ of ropivacaine in combination with ropivacaine at different doses of dexmedetomidine so that the minimum effective dose of local anesthetic concentration can not only ensure an anesthetic effect but also decrease the degree of adverse reactions or even reduce the occurrence of adverse reactions, which may be more beneficial to patients.

## 5 Limitations

(1) Although the benefits of dexmedetomidine are more easily manifested in major surgery, surgery with large hemodynamic fluctuation was not selected in this study. This study aimed to explore the effect of different doses of dexmedetomidine administration on the perioperative hemodynamics and anesthesia effect of patients, and there are too many uncontrollable factors in major surgery, which is easy to cause interference. In future, further experiments can be conducted in the population of major surgery to observe the effects of epidural infusion of different doses of dexmedetomidine on the perioperative hemodynamics and anesthetic effect of patients. (2) Although this experiment has verified that the epidural infusion of 0.75 μg/kg dexmedetomidine has less impact on the perioperative circulation of patients and better anesthetic effect compared to 0.25 μg/kg and 0.5 μg/kg dexmedetomidine, the slowing of heart rate and prolongation of motor block time caused by epidural infusion should not be underestimated, and this adverse reaction is considered to be associated with the reduction of ED_95_ of ropivacaine by dexmedetomidine. Currently, it is not clear that the ED_95_ of ropivacaine at different doses of dexmedetomidine requires further experimental research. (3) The concentration of dexmedetomidine in perioperative cerebrospinal fluid and blood was not measured in our study, and it is unclear whether the pharmacodynamics and pharmacokinetics of epidural dexmedetomidine are related to the present study results, and further research is needed.

## 6 Conclusion

Based on these findings, 0.5 μg/kg dexmedetomidine is more suitable as an epidural anesthetic adjuvant with 0.59% ropivacaine. However, further research is needed to determine the optimal local anesthetic concentration of ropivacaine combined with higher doses (such as 0.75 μg/kg) of dexmedetomidine for epidural anesthesia.

## Data availability statement

The original contributions presented in the study are included in the article/supplementary material, further inquiries can be directed to the corresponding author.

## Ethics statement

The studies involving humans were approved by Medical Ethics Committee of the Affiliated Hospital of North Sichuan Medical College, registration number: ChiCTR2200060619. The studies were conducted in accordance with the local legislation and institutional requirements. The participants provided their written informed consent to participate in this study.

## Author contributions

SZ: Conceptualization, Data curation, Formal analysis, Methodology, Resources, Software, Supervision, Visualization, Writing – original draft, Writing – review & editing. XL: Funding acquisition, Methodology, Project administration, Resources, Software, Supervision, Validation, Visualization, Writing – original draft, Writing – review & editing. HX: Data curation, Investigation, Methodology, Project administration, Supervision, Writing – review & editing. QY: Funding acquisition, Resources, Supervision, Visualization, Writing – review & editing. ZL: Funding acquisition, Resources, Software, Writing – review & editing. FW: Conceptualization, Data curation, Formal analysis, Funding acquisition, Investigation, Methodology, Project administration, Resources, Software, Supervision, Validation, Visualization, Writing – original draft, Writing – review & editing.

## Appendix

1. Ramsay Sedation Score (18): (1) Awake but not quiet, irritable is counted as 1 point. (2) Basic wakefulness, quiet cooperation is credited as 2 points. (3) Drowsiness, being able to follow instructions is counted as 3 points. (4) Sleep state, but arousal is counted as 4 points. (5) A light shake wakes up, but unresponsive is counted as 5 points. (6) Deep sleep, shoulder shaking and unresponsive is counted as 6 points. A Ramsay score greater than 4 points as excessive sedation.
2. Degree of forgetting: divided into (1) No forgetting: can correctly recall the sound of instrument operation, medical staff dialogue, or surgical discomfort. (2) Incomplete forgetting: can be partially recalled after prompting. (3) Complete forgetting: cannot be recalled after prompting.
3. Bromage lower limb motor block degree score (19): within 5 min after the administration of local anesthetics, the lower limb movement was determined every minute and then every 3 min until the beginning of the surgery. (1) 1 point for a complete block of movement. (2) 2 points for movement with only the ankle off energy conservation. (3) 3 points for movement with only the knee joint movable. (4) 4 points for lifting the leg up but not holding it up. (5) 5 points for lifting the leg up and holding it up for more than 10s. (6) 6 points for complete absence of movement block. The number of cases with Bromage grade < 2 points, 2–4 points, and > 4 points was recorded.
4. VAS score (16): allows the patient to mark the 10 cm horizontal line according to self-feeling: (1) Painless (0 points). (2) Mild pain, tolerable (1–3 points). (3) Pain interferes with sleep and is tolerable (4–6 points). (4) Unbearable pain, affecting appetite and sleep (7–10 points).
5. Anesthesia Satisfaction Scale (20): On postoperative day 1, patients were asked whether or not they were satisfied with their postoperative pain control and to score their overall satisfaction from 0 (totally dissatisfied) to 10 (totally satisfied).
6. Myocardial oxygen consumption (MVO_2_) was calculated using the formula (21): heart rate × systolic blood pressure binomial product (RPP) to roughly estimate myocardial oxygen consumption.
